# Development and Maintenance of the Gut-Associated Lymphoid Tissue (Galt): the Roles of Enteric Bacteria and Viruses

**DOI:** 10.1155/1998/68382

**Published:** 1998

**Authors:** John J. Cebra, Sangeeta Bhargava Periwal, Gwen Lee, Fan Lee, Khushroo E. Shroff

**Affiliations:** 1 Department of Biology University of Pennsylvania Philadelphia PA 19104-6018 USA; 2 Department of Biology University of Pennsylvania 415 South University Avenue Philadelphia PA 19104-6018 USA

**Keywords:** Enteric viruses, gut-associated lymphoid tissue (GALT), gut commensal bacteria, IgA responses in gut, intraepithelial leukocytes, Peyer's patches

## Abstract

GALT can be subdivided into several compartments: (a) Peyer's patches (PP); (b) lamina propria (LP); and (c) intraepithelial leukocyte (IEL) spaces. The B-cell follicles of PP are quiescent in neonatal and germ-free (GF) adult mice. Germinal centers (GC), including sIgA^+^ blasts, appear in the B follicles of formerly GF adult mice about 10-14 days after monoassociation with various gut commensal bacteria. The GC wax and wane over about a 3-week period, although the bacterial colonizers remain in the gut at high density. Neonatal mice, born of conventionally reared (CV), immunocompetent mothers, display GC reactions in PP postweaning, although pups of SCID mothers display precocious GC reactions at about 14 days of life. Normally, gut colonization of neonates with segmented filamentous bacteria (SFB) leads to explosive development of IgA plasmablasts in LP shortly after weaning. Commensal gut bacteria and the immunocompetency of mothers also appears to control the rate of accumulation of primary B cells from “virgin” B cells in neonates.

Enteric reovirus infection by the oral route can cause the activation of CD8^+^ T cells in the interfollicular regions of PP and the appearance of virus-specific precursor cytotoxic T lymphocytes (pCTL) in the IEL spaces. Such oral stimulation can also lead to “activation” of both CTL and natural killer (NK) cells in the IEL spaces. More normally, colonization of the gut with SFB also leads to similar activations of NK cells and “constitutively” cytotoxic T cells.


*Developmental Immunology*, 1998, Vol. 6, pp. 13–18
Reprints available directly from the publisher
Photocopying permitted by license only

© 1998 OPA (Overseas Publishers Association)
N.V. Published by license under the
Harwood Academic Publishers imprint,
part of the Gordon and Breach Publishing Group
Printed in Malaysia

### Development and Maintenance of the Gut-Associated Lymphoid Tissue (GALT): The Roles of Enteric Bacteria and Viruses

JOHN J. CEBRA*, SANGEETA BHARGAVA PERIWAL, GWEN LEE, FAN LEE and KHUSHROO E. SHROFF

*Department of Biology, University of Pennsylvania, Philadelphia, PA 19104-6018*

(*Received 16 August 1996*; *Accepted 14 August 1997*)

GALT can be subdivided into several compartments: (a) Peyer's patches (PP); (b) lamina propria (LP); and (c) intraepithelial leukocyte (IEL) spaces. The B-cell follicles of PP are quiescent in neonatal and germ-free (GF) adult mice. Germinal centers (GC), including sIgA$^+$ blasts, appear in the B follicles of formerly GF adult mice about 10-14 days after monoassociation with various gut commensal bacteria. The GC wax and wane over about a 3-week period, although the bacterial colonizers remain in the gut at high density. Neonatal mice, born of conventionally reared (CV), immunocompetent mothers, display GC reactions in PP postweaning, although pups of SCID mothers display precocious GC reactions at about 14 days of life. Normally, gut colonization of neonates with segmented filamentous bacteria (SFB) leads to explosive development of IgA plasmablasts in LP shortly after weaning. Commensal gut bacteria and the immunocompetency of mothers also appears to control the rate of accumulation of primary B cells from "virgin" B cells in neonates.
Enteric reovirus infection by the oral route can cause the activation of CD8$^+$ T cells in the interfollicular regions of PP and the appearance of virus-specific precursor cytotoxic T lymphocytes (pCTL) in the IEL spaces. Such oral stimulation can also lead to "activation" of both CTL and natural killer (NK) cells in the IEL spaces. More normally, colonization of the gut with SFB also leads to similar activations of NK cells and "constitutively" cytotoxic T cells.

*Keywords:* Enteric viruses, gut-associated lymphoid tissue (GALT), gut commensal bacteria, IgA responses in gut, intraepithelial leukocytes, Peyer's patches

### INTRODUCTION

### Lymphoid Tissues of GALT

GALT includes both organized lymphoid compartments, consisting of PP, regional lymphatics, and mesenteric lymph nodes (MLN), and dispersed lymphoid cells in the IEL spaces and the gut LP (Owen and Jones, 1974; Cerf-Bensussan and Guy-Grand, 1991). The PP consist of a single-layer cluster of B-cell follicles, divided by T-cell-rich wedges, unevenly distributed in the wall of the small intestine. A

*Corresponding author. Present address: Department of Biology, University of Pennsylvania, 415 South University Avenue, Philadelphia, PA 19104-6018.

specialized follicle-associated epithelium (FAE) overlies these clusters, forming a relatively mucus-free "dome" among the absorptive villi and their interspersed crypts. Among the cells of FAE are specialized M (microfold) cells, which actively pinocytose or endocytose droplets and particles from the gut lumen Owen and Jones, 1974). These M cells serve as "afferent lymphatics" for PP, delivering antigens (Ags) and pathogens to the underlying, organized lymphoid tissue (Wolf et al., 1981; Jones et al., 1994). The B-cell follicles in PP of CV mammals are not typically quiescent (primary), as in lymph nodes or spleen, but rather display continuous GC reactions and are chronically activated (secondary). Presumably, these GC reactions are constantly driven by the environmental Ags delivered via M cells. The GC in PP of mice typically bind high levels of peanut agglutinin (PNA—a marker for the B blasts) and contain a preponderance of dividing, IgA-expressing B blasts (Lebman et al., 1987; Weinstein et al., 1991). Surface IgD-bearing B cells (primary B cells) are confined to the recirculating population comprising the surrounding mantle zone.

### Lymphocyte Recirculation from and to GALT

Among the B cells leaving PP via efferent lymphatics are $IgA^+$ B cells, generated and selected for survival in GC (Craig and Cebra, 1971; Lebman et al., 1987). These pass through MLN, where some are maturing to IgA plasma blasts (McWilliams et al., 1975). Eventually, these $IgA^+$ B cells are found in thoracic duct lymph (Pierce and Gowans, 1975) and blood, having developed a homing propensity to exit into and accumulate in mucosal tissues via transit of small venules (Husband, 1982). Many of the $IgA^+$ plasmablasts generated in PP eventually accumulate and secrete their IgA Abs in the gut LP.

At least one T-cell subset that can contribute to gut immunity ($CD8^+$ CTL) can be generated by Ag-stimulation in the interfollicular regions of PP (London et al., 1987, 1990). Some of these emigrate to the IEL spaces, where they selectively lodge and can function as one subset of CTL among other NK and $CD8^+$ T cells from other sources (Cuff et al., 1993).

### Roles of IgA Abs and T Lymphocytes in GALT

Dimeric IgA Abs, produced locally by IgA plasma cells in gut LP, are actively transported via poly Ig-receptors (pIg-R) into and through gut epithelial cells, especially crypt cells (Mostov et al., 1980). During exocytosis into crypt and gut lumen, the pIg-R is cleaved and a portion (secretory component) remains complexed to IgA dimer. This "secretory" IgA can function as a "blocking" or "neutralizing" Ab in the gut lumen, preventing the attachment or fucntioning of viral or bacterial pathogens or enterotoxins onto or in target cells, usually gut epithelial cells (Ogra and Karzon, 1969; Merchant et al., 1991). Very recently, an intriguing protective activity has been suggested for IgA dimer present in LP below the mucosal epithelium or within epithelial cells: that this IgA dimer can complex with Ags after their uptake by epithelial cells and even after their translocation of these cells (Mazanec et al., 1992). The result could be interference with intracellular viral or bacterial replication and/or "flushing out" or "rejection" of the Ag/IgA dimer complex via the normal pIg-R mediated transport mechanisms.

Direct evidence for the functioning of effector CTL in the IEL spaces has been difficult to obtain. However, their abundance—one per five epithelial cells Cerf-Bensussan and Guy-Grand, 1991)—and selective accumulation in these spaces via special adhesion molecules for ligands on enterocytes (Cepek et al., 1994) support a role at these sites. We find that pCTL, generated in PP, can populate the IEL spaces, and that these can protect neonatal mice against an oral infection with Type 3 reovirus, which ordinarily leads to a fatal meningoencephalitis (Cuff et al., 1991, 1993).

### RESULTS AND DISCUSSION

### The Use of Murine Models to Analyze the Roles of Enteric Viruses and Bacteria in Driving the Development of the Mucosal Immune System

In order to circumvent the normally constant stimuli from environmental Ags in the gut lumen, we used mice-raised GF in flexible-frame, Trexler isolation bubbles. Although such mice are not "Ag-free," since they receive autoclaved food containing foreign macromolecules, their GALT exhibits a marked depression of the physiologically normal state of activation and hypertrophy: B-cell follicles of PP are primary and lack GC, gut lumen LP contains few IgA plasma cells (<5% of "normal"), and both $CD4^+$ and $CD8^+$ T cells are mostly in a low state of activation.

Initially, we used Type 1 reovirus to probe the mucosal immune system (London et al., 1987). When orally administered, this virus causes a transient infection of the gut epithelium of adult, immunocompetent mice without any overt clinical symptoms. We compared oral versus footpad administration of reovirus. GC reactions occurred in the PP of GF mice and LN of CV mice, respectively. These GC reactions similarly waxed and waned over about 4 weeks, reaching a maximum at about 10-14 days (Weinstein and Cebra, 1991). At 12-14 days postinfection, the infectious virus is completely resolved. We compared the specific Ab responses in PP versus LN using a tissue fragment culture assay that we developed (Logan et al., 1991). This assay reflects the status of Ag-specific priming and Ig isotype potential by Ab secreted in culture over 8 days, after removing lymphoid tissue from mice at various times after local infection *in vivo*. Although the time course of the GC reactions were similar in PP versus LN after local infection, the local Ab responses differed markedly: Following IgM Ab responses detectable in both tissue fragment cultures set up 3 days after *in vivo* infection, only the PP cultures expressed IgA Abs, beginning 5 days after *in vivo* infection, whereas only LN cultures expressed IgG1 Abs when set up at about the same time after *in vivo* infection.

Another major difference between the outcomes of local infection at the two different sites is that a secondary viral challenge at the original site results in an appreciably greater Ab response—and relatively more IgG1 Abs—in LN, but a considerably lesser Ab response in PP, compared with the initial responses after primary infection (Weinstein and Cebra, 1991). From these observations, we conclude that (1) chronic GC reactions in PP are not constitutive, but must be consecutively induced by overlapping gut mucosal exposures to Ag; (2) the preferred isotype switching to IgA expression by dividing B cells in PP is a consequence of their special microenvironment, rather than their chronically activated state; and (3) IgA Abs, secreted into the gut lumen after primary gut mucosal infection, continue to block uptake of viral Ag upon subsequent oral/mucosal challenge, thus attenuating the effective secondary stimulus.

Based on these observations, we wondered whether chronic gut colonization of formerly GF mice with an enteric bacteria would provoke only a transient GC reaction in PP or present a continuing stimulus that would maintain chronic GC reactions. How does the GALT of mammals respond to commensal enterics? We monoassociated GF mice with a single species of Gram-negative bacilli, *Morganella morganii*, by oral inoculation (Shroff et al., 1995). Surprisingly, to us, we observed about the same time course of GC reactions in PP—by incorporation of BrdU for dividing cells, appearance of $PNA^+$, $IgA^+$ B blasts—after colonization with *M. morganii* as we had observed following acute gut mucosal reovirus infection (Weinstein and Cebra, 1991). By days 30-50 postcolonization, the GC reactions waned and were resolved, although the now quiescent B follicles maintained more $IgA^+$ B cells and $CD4^+$ T cells scattered throughout the follicles and in the dome region than were found in PP of GF mice prior to colonization. Tissue fragment cultures of PP and small intestine (SI) showed a maximal output of IgA Abs 10-70 days postcolonization and a slow decline thereafter during nearly 1 year of monitoring. The potentials of the fragment cultures reflected the appearance and maintenance of specific IgA Ab-secreting plasma cells in gut LP. Although some of the enterics translocated to MLN and spleen, systemic bacteria were completely cleared within weeks. The commensals were then confined to the gut, where they continued to maintain themselves at high densities ($\approx 10^9$/gram feces) for at least 1 year, although they exhibited a "coating" with the host's IgA within 5-6 days after colonization, which was then also maintained. We concluded that the mammalian host does make a humoral immune response to commensal bacteria, but that this response is self-limiting, probably due to continuous, low-level production and secretion of IgA Abs, which act to exclude both bacteria and their Ags from effective, continuing stimulation of PP. These observations further support the hypothesis that the chronic GC reactions ordinarily occurring in CV mammals must be due to consecutive, overlapping gut mucosal stimuli provided by changing and/or novel environmental Ags.

### External Factors that Influence the Development of the Humoral Mucosal and Systemic Immune System of Neonates

It has been known for many years that neonatal mice do not express "natural" IgA Abs reactive with gut commensal bacteria and that their gut LP is essentially devoid of IgA plasma cells. Ordinarily, neonatal mice first express appreciable natural IgA in their gut secretions around the time of weaning—days 21-28 of life. We wondered whether such neonates were capable of displaying a specific IgA Ab response earlier, upon challenge of their gut mucosa with a foreign, nonenvironmental Ag. Using oral inoculation with Type 1 reovirus and both PP and SI fragment cultures, we showed that neonatal mice could initiate an active humoral mucosal immune response, including predominantly IgA Abs, at least as early as day 10 of life (Kramer and Cebra, 1995a). We also observed, in the course of these studies, that day 10 neonates, orally infected with enteric reovirus, also developed about a 20-fold greater expression of natural IgA in their gut mucosa than of demonstrably virus-specific IgA Abs. We wondered whether maternal immune influences ordinarily affected the typical delay in "spontaneous" development of natural IgA in neonates until weaning. In order to examine this possibility, we made reciprocal matings of immunocompetent mice with severe-combined immunodeficient (SCID) mice. The $F_1$ offspring were all expected to be immunocompetent and to only differ by whether their dam was SCID or immunocompetent. We found that both sets of $F_1$ pups responded equally well to oral reovirus challenge, unless their mother was immunocompetent and had previously been orally inoculated with reovirus. Swapping litters and foster nursing indicated that a mucosally immune nurse mother was essential to prevent active mucosal immunization of the pups during suckling. Furthermore, in the absence of deliberate oral inoculation with reovirus, $F_1$ pups from the reciprocal crosses differed in the time of spontaneous development of natural IgA Abs, GC reactions in PP, and the appearance of IgA plasma cells in gut LP: $F_1$ pups born to and nursed on SCID dams exhibited a precocious development of these features by days 14-16 of life, whereas $F_1$ pups from immunocompetent dams showed the typical delay in these changes until weaning (days 21-28) (Kramer and Cebra, 1995b). We were able to correlate the "delayed" development of active natural IgA responsiveness in the gut of neonates with the passively acquired maternal IgA content of the stomach and the "coating" of most elements of the neonatal gut flora with this maternal IgA. Finally, oral infection of neonates from nonimmune (to reovirus) immunocompetent dams with reovirus appears to overcome the blocking effects of suckled maternal IgA, reactive with the gut bacteria of neonates.

We wondered whether suckled maternal Abs versus bacterial Ags/polyclonal mitogens might also regulate other aspects of B-cell development in neonates. Monroe and colleagues have shown a delay until about 18-28 days of life in the competence of splenic neonatal B cells to respond *in vitro* to cross-linking of their sIg Ag receptors with $Fab'_2$ anti-mouse IgM (Yellen-Shaw and Monroe, 1992; Monroe et al., 1993). Neonatal and adult B cells are about equally responsive to mitogenic stimulation with LPS. This change in responsiveness is a functional correlate of the "virgin" to "primary" B-cell transition. Examination of *in vitro* responsiveness of neonatal, splenic B cells from $F_1$ pups of the previously described, reciprocal crosses also show that pups born and nursed by or only foster suckled by SCID dams exhibited a precocious transition virgin to primary B cells. We suggest that bacterial LPS, or some other bacterial Ags, may play a role in driving this transition and that suckled maternal Abs versus these products of the gut flora may partially block their uptake and dissemination.

TABLE I Stimulation of Germinal-Center Reactions and CD4+ T-Cell Activation in Peyer's Patches Following Colonization in Germ-Free C3H Mice with Segmented Filamentous Bacteria[a]

| Mice | % PP Lymphocytes | | % PP CD4+ cells |
|---|---|---|---|
| | PNA+ | sIgA+ | CD45RB$^{high}$ |
| GF | 4.9 | 1.2 | 65 |
| GF, 6 day pi | 4.2 | 1.5 | 60 |
| GF, 14 day pi | 19.0 | 3.0 | 56 |
| GF, 28 day pi | 16.0 | 6.5 | 54 |
| GF, 50 day pi | 9.4 | 5.5 | 38 |
| CV adults | 15.0 | 3.8 | 33 |
| CV adults | 20.0 | 4.1 | 46 |

[a]PNA+ = peanut agglutinin binding; sIgA+ = surface IgA positive; GF = germ-free; p.i. = postinfection (colonization of the gut); CV = conventionally reared (normal, complex gut flora).

### Can One Identify Members of the Gut Bacterial Flora that Play a Significant Role in Driving the Development of the GALT?

A prominent group of bacteria in the normal gut flora of many animals was identified as early as 1849 (Leidy, 1849) as "jointed threads" (Arthromitis) or "segmented filamentous bacteria" (SFB). These SFB are Gram-positive, spore-forming, obligate anaerobes that have never been cultured *in vitro*. Recently, SFB have been "cloned" by limiting dilution of spore suspensions into GF mice Klaasen et al., 1991). Such monoassociated, formerly GF mice show the rapid development of a population of IgA plasma cells in gut LP and of natural IgA in gut secretions (Klaasen et al., 1993). A "cocktail" of other, incompletely characterized members of the commensal gut flora, lacking SFB, caused little stimulation of the development of GALT in formerly GF mice.

We have followed the development of GC reactions, including prominent expansion of IgA+ B blasts, in formerly GF mice colonized with SFB. Table 1 shows that, over the first 50 days following colonization, GC reactions wax and begin to wane with a time course similar to that observed following monoassociation of GF mice with *M. morganii*. We are now examining the specificity of this response and the persistance of IgA plasma cells in the gut LP of these colonized mice. Since SFB appears reather explosively in the intestine of neonatal mice at the time of weaning, and then expands further to become the dominant gut bacteria over the next 3-4 months, it is tempting to suggest a major role for it in driving the development of the humoral mucosal immune system. Of particular interest would be to determine whether SFB plays an effective role in the populating of gut LP with IgA plasma cells derived from the B1 lineage (see Bos et al., 1996).

Recently, we have begun to assess whether gut colonization with SFB may also contribute to the activation of the cellular mucosal immune system. Table 1 also shows that such colonization of GF mice results in an overall shift in the subpopulations of CD4+ T cells of PP from a dominance of CD45RB$^{high}$ cells to a preeminence of CD45RB$^{low}$ cells. Such shifts in phenotype have been associated with T-cell priming and activation. We have also examined two cellular elements of the IEL population: NK cells and CD8+ T cells. Preliminary findings are that GF mice have much lower levels of NK activity and "constitutively cytotoxic" CD8+ T-cell activity in their IEL populations than CV-reared mice. By about 50-60 days following colonization of GF C3H mice with SFB, IEL populations show a significant rise in both kinds of cytotoxic activity, and these functional changes are accompanied by shifts in subsets of both NK and CD8+ T cells.

### *Acknowledgements*

These studies were supported by grants AI-35936, AI-23970, and AI-37108 from the National Institute for Allergy and Infectious Diseases.

### *References*

Bos N.A., Bun J.C.A.M., Popma S.H., Cebra E.R., Deenen G.J., van der Cammen J.F., Kroese F.M.G., and Cebra J.J. (1996). Monoclonal IgA derived from peritoneal B cells is encoded both by germline and somatically mutated Vh genes and is reactive with commensal bacteria. Infect. Immun. **64**: 616-623.

Cepek K.L., Shaw S.K., Parker C.M., Russell G.J., Morrow J.S., Rimm D.L., and Brenner M.B. (1994). Adhesion between epithelial cells and T lymphocytes mediated by E-cadherin and the alpha E beta 7 integrin. Nature **372(6502)**: 190-193.

Cerf-Bensussan N., and Guy-Grand D. (1991). Intestinal intra-epithelial lymphocytes. Gastroenterol. Clinics North Amer. **20**: 549-575.

Craig S.W., and Cebra J.J. (1971). Peyer's patches: An enriched source of precursors for IgA-producing immunocytes in the rabbit. J. Exp. Med. **134**: 188-200.

Cuff C.F., Cebra C.K., Lavi E., Molowitz E.H., Rubin D.H., and Cebra J.J. (1991). Protection of neonatal mice from fatal reovirus infection by immune serum and gut derived lymphocytes. In Immunology of Milk and the Neonate, Mestecky J., et al., Eds. (New York: Plenum Press), pp. 307-315.

Cuff C.F., Cebra C.K., Rubin D.H., and Cebra J.J. (1993). Developmental relationship between cytotoxic $\alpha/\beta$ T cell receptor-positive intraepithelial lymphocytes and Peyer's patch lymphocytes. Eur. J. Immunol. **23**: 1333-1339.

Husband A.J. (1982). Kinetics of extravasation and redistribution of IgA-specific antibody-containing cells in the intestine. J. Immunol. **128**: 1355-1359.

Jones B.D., Ghori N., and Falkow S. (1994). Salmonella typhimurium initiates murine infection by penetrating and destroying the specialized epithelial M cells of the Peyer's patches. J. Exp. Med. **180**: 15-23.

Klaasen H.L.B.M., Koopman J.P., Van den Brink M.E., Van Wezel H.P.N., and Beynen A.C. (1991). Mono-association of mice with non-cultivable, intestinal, segmented filamentous bacteria. Arch. Microbiol. **156**: 148-151.

Klaasen H.L.B.M., Van der Heijden P.J., Stok W., Poelma F.G.J., Koopman J.P., Van den Brink M.E., Bakker M.H., Eling W.M.C., and Beynen A.C. (1993): Apathogenic, intestinal, segmented, filamentous bacteria stimulate the mucosal immune system of mice. Infect. Immunol. **61**: 303-306.

Kramer D.R., and Cebra J.J. (1995a). Role of maternal antibody in the induction of virus specific and bystander IgA responses in Peyer's patches of suckling mice. Int. Immunol. **7**: 911-918.

Kramer D.R., and Cebra J.J. (1995b). Early appearance of "natural" mucosal IgA responses and germinal centers in suckling mice developing in the absence of maternal antibodies. J. Immunol. **154**: 2051-2062.

Lebman D.A., Griffin P.M., and Cebra J.J. (1987). Relationship between expression of IgA by Peyer's patch cells and functional IgA memory cells. J. Exp. Med. **166**: 1405-1418.

Leidy J. (1849). On the existence of entophyta in healthy animals as a natural condition. Proc. Acad. Natl. Sci. Phila **4**: 225-233.

Logan A.C., Chow K.-P.N., George A., Weinstein P.D., and Cebra J.J. (1991). Use of Peyer's patch and lymph node fragment cultures to compare local immune responses to Morganella morganii. Infect. Immunol. **59**: 1024-1031.

London S.D., Cebra-Thomas J.A., Rubin D.H., and Cebra J.J. (1990). CD8 lymphocyte subpopulations in Peyer's patches induced by reovirus serotype 1 infection. J. Immunol. **144**: 3187-3194.

London S.D., Rubin D.H., and Cebra J.J. (1987). Gut mucosal immunization with reovirus serotype 1/L stimulates virus-specific cytotoxic T cell precursors as well as IgA memory cells in Peyer's patches. J. Exp. Med. **165**: 830-847.

Mazanec M.B., Kaetzel C.S., Lamm M.E., Fletcher D., and Nedrud J.G. (1992). Intracellular neutralization of virus by immunoglobulin A antibodies. Proc. Natl. Acad. Sci. USA **89**: 6901-6905.

McWilliams M., Phillips-Quagliata J.M., and Lamm M.E. (1975). Characteristics of mesenteric lymph node cells homing to gut-associated lymphoid tissue in syngeneic mice. J. Immunol. **115**: 54-58.

Merchant A.A., Groene W.S., Cheng E.H., and Shaw R.D. (1991). Murine intestinal antibody response to heterologous rotavirus infection. J. Clin. Microbiol. **29**: 1693-1701.

Monroe J., Yellen-Shaw A., and Seyfert V. (1993). Molecular basis for unresponsiveness and tolerance induction in immature stage B lymphocytes. Adv. Molec. Cell. Immunol. **1B**: 1-32.

Mostov K.E., Kraehenbuhl J.P., and Blobel G. (1980). Receptor-mediated transcellular transport of immunoglobulin: Synthesis of secretory component as multiple and larger transmembrane forms. Proc. Natl. Acad. Sci. USA **77**: 7257-7261.

Ogra P.L., and Karzon D.T. (1969). Distribution of poliovirus antibody in serum, nasopharynx and alimentary tract following segmental immunization of lower alimentary tract with poliovaccine. J. Immunol. **102**: 1423-1430.

Owen R.L., and Jones A.L. (1974). Epithelial cell specialization within Peyer's patches: An ultrastructural study of intestinal lymphoid follicles. Gastroenterology **66**: 189-203.

Pierce N.F., and Gowans J.L. (1975). Cellular kinetics of the intestinal immune response to cholera toxoid in rats. J. Exp. Med. **142**: 1550-1563.

Shroff K.E., Meslin K., and Cebra J.J. (1995). Commensal enteric bacteria engender a self-limiting humoral mucosal immune response while permanently colonizing the gut. Infect. Immunol. **63**: 3904-3913.

Weinstein P.D., and Cebra J.J. (1991). The preference for switching to IgA expression by Peyer's patch germinal center B cells is likely due to the intrinsic influence of their microenvironment. J. Immunol. **147**: 4126-4135.

Weinstein P.D., Schweitzer P.A., Cebra-Thomas J.A., and Cebra J.J. (1991). Molecular genetic features reflecting the preference for isotype switching to IgA expression by Peyer's patch germinal center B cells. Int. Immunol. **3**: 1253-1263.

Wolf J.L., Rubin D.H., Finberg R., Kauffman R.S., Sharpe A.H., Trier J.S., and Fields B.N. (1981). Intestinal M cells: A pathway for entry of reovirus into the host. Science **212**: 471-472.

Yellen-Shaw A., and Monroe J. (1992). Differential unresponsiveness of immature- and mature-stage murine B cells to anti-IgM reflects both FcR-dependent and -independent mechanisms. Cell. Immunol. **145**: 339-350.

